# Individual and poly-substance use and condomless sex among HIV-uninfected adults reporting heterosexual sex in a multi-site cohort

**DOI:** 10.1186/s12889-021-12026-7

**Published:** 2021-11-04

**Authors:** R. J. Fredericksen, B. M. Whitney, E. Trejo, R. M. Nance, E. Fitzsimmons, F. L. Altice, A. W. Carrico, C. M. Cleland, C. Del Rio, A. Duerr, W. M. El-Sadr, S. Kahana, I. Kuo, K. Mayer, S. Mehta, L. J. Ouellet, V. M. Quan, J. Rich, D. W. Seal, S. Springer, F. Taxman, W. Wechsberg, H. M. Crane, J. A. C. Delaney

**Affiliations:** 1 UW Center for AIDS Research, Harborview Medical Center, 325 Ninth Avenue, Box 359931, Seattle, WA 98104-2499 USA; 2 Yale University AIDS Program, 135 College Street, Suite 323, New Haven, CT 06510-2283 USA; 3 Division of Prevention Science and Community Health, University of Miami, 1120 NW 14th St, Miami, FL 33136 USA; 4 Center for Drug Use and HIV Research, NYU School of Global Public Health, 665 Broadway, 11th Floor, New York, NY 10012 USA; 5 Rollins School of Public Health, Emory University, 1518 Clifton Road, NE Room 7011, Atlanta, GA 30322 USA; 6 Fred Hutchinson Cancer Research Center, HIV Vaccine Trials Network, Box 358080 (LE 500), Seattle, WA 98109 USA; 7 Mailman School of Public Health, Columbia University, 722 West 168th Street, 13th floor, New York, NY 10032 USA; 8 National Institute on Drug Abuse, 6001 Executive Blvd, Rockville, Maryland 20852 USA; 9 Department of Epidemiology and Biostatistics, Milken Institute School of Public Health, George Washington University, 950 New Hampshire Ave NW #2, Washington, DC 20052 USA; 10 The Fenway Institute, 1340 Boylston Street, Boston, MA 02215 USA; 11 Johns Hopkins University, Bloomberg School of Public Health, 615 N. Wolfe Street, Baltimore, Maryland 21205 USA; 12 School of Public Health, University of Illinois at Chicago, 1603 W. Taylor St, Chicago, IL USA; 13 Department of Epidemiology, Bloomberg School of Public Health, Johns Hopkins University, 615 N. Wolfe Street, Baltimore, Maryland 21205 USA; 14 Center for Prisoner Health and Human Rights, Immunology Center, The Miriam Hospital, Warren Alpert Medical School, Brown University, 1125 North Main St, Providence, RI 02904 USA; 15 Department of Global Community Health and Behavioral Sciences, School of Public Health and Tropical Medicine, Tulane University, 1440 Canal St, Suite 2200, New Orleans, LA 70112 USA; 16 Department of Internal Medicine, School of Medicine, Yale University, 135 College Street, New Haven, CT 06510 USA; 17 Center for Advancing Correctional Excellence, Institute of Biohealth Innovation, George Mason University, 4461 Rockfish Creek Lane, Fairfax, VA 22030 USA; 18 Department of Health Policy and Management, Gillings School of Public Health, University of North Carolina Chapel Hill, 135 Dauer Dr, Chapel Hill, NC 27599 USA; 19 College of Pharmacy, University of Manitoba, Apotex Centre, 750 McDermot Avenue, Winnipeg, Manitoba R3E 0T5 Canada

**Keywords:** Substance use, Condom use

## Abstract

**Background:**

We analyzed the association between substance use (SU) and condomless sex (CS) among HIV-negative adults reporting heterosexual sex in the Seek, Test, Treat, and Retain (STTR) consortium. We describe the impact of SU as well as person/partner and context-related factors on CS, identifying combinations of factors that indicate the highest likelihood of CS.

**Methods:**

We analyzed data from four US-based STTR studies to examine the effect of SU on CS using two SU exposures: 1) recent SU (within 3 months) and 2) SU before/during sex. Behavioral data were collected via 1:1 or self-administered computerized interviews. Adjusted individual-study, multivariable relative risk regression was used to examine the relationship between CS and SU. We also examined interactions with type of sex and partner HIV status. Pooled effect estimates were calculated using traditional fixed-effects meta-analysis. We analyzed data for recent SU (*n* = 6781; 82% men, median age = 33 years) and SU before/during sex (*n* = 2915; 69% men, median age = 40 years).

**Results:**

For both exposure classifications, any SU other than cannabis increased the likelihood of CS relative to non-SU (8–16%, *p*-values< 0.001). In the recent SU group, however, polysubstance use did not increase the likelihood of CS compared to single-substance use. Cannabis use did not increase the likelihood of CS, regardless of frequency of use. Type of sex was associated with CS; those reporting vaginal and anal sex had a higher likelihood of CS compared to vaginal sex only for both exposure classifications (18–21%, *p* < 0.001). Recent SU increased likelihood of CS among those reporting vaginal sex only (9–10%, *p* < 0.001); results were similar for those reporting vaginal and anal sex (5–8%, *p* < 0.01). SU before/during sex increased the likelihood of CS among those reporting vaginal sex only (20%; p < 0.001) and among those reporting vaginal and anal sex (7%; *p* = 0.002). Single- and poly-SU before/during sex increased the likelihood of CS for those with exclusively HIV-negative partners (7–8%, *p* ≤ 0.02), and for those reporting HIV-negative and HIV-status unknown partners (9–13%, *p* ≤ 0.03).

**Conclusion:**

Except for cannabis, any SU increased the likelihood of CS. CS was associated with having perceived HIV-negative partners and with having had both anal/vaginal sex.

**Supplementary Information:**

The online version contains supplementary material available at 10.1186/s12889-021-12026-7.

## Background

Sexually transmitted infections (STIs) including HIV disproportionately affect socially and economically marginalized populations [1] including African-Americans, Latinos, men who have sex with men (MSM), including bisexual men that also have sex with women, transgender women, and those currently or formerly incarcerated [2–9]. Beyond abstinence, condoms are the most effective means of preventing most STIs. The use of intoxicants prior to sex is a well-known barrier to condom use, [10–12] and substance use (SU) is common among many of the populations at greatest risk for contracting STIs/HIV [13–15].

Much heterogeneity exists among studies in the strength of associations between SU and condomless sex (CS), particularly between those studies with different methodologies and recall periods [16–19]. The literature suggests general associations between alcohol consumption, CS, and HIV risk, though the studies do not establish a causal relationship. More rigorous multiple event-level analyses have provided the temporal sensitivity to assess causality, from which there have been mixed findings [17, 20–23]. Event-level analyses for CS in the context of substances other than alcohol have varied by substance: while methamphetamine use, cocaine, and illicit opioids have been associated with CS [22–26], effects for cannabis use have been mixed [20, 21, 23, 27–30].

Additionally, the association of SU with CS has been found to vary substantially by gender and partner characteristics [31], HIV status [32], nature of relationship (i.e. primary vs. casual vs. transactional) [33], number of substances used [34], combination of substances used [32, 35], and pattern of substance use [10, 33].

Previous studies of condom use behavior in the context of substance use among HIV-uninfected adults reporting heterosexual sex in the U.S. have not specified type of sex (e.g., anal vs. vaginal), have been substance-specific, or have had limited sample size. To better understand the relationship between SU and CS, we analyzed CS among a large cohort of HIV-uninfected adults reporting heterosexual sex in the context of SU, derived from the harmonized dataset of the National Institute of Drug Abuse (NIDA) Seek, Test, Treat, and Retain (STTR) HIV-prevention and treatment research initiative [36]. We describe the association between substance-related, person/partner related, and context-related factors and condom use. In the process we identify the combinations of factors that indicate the highest risk of CS, and hence potential for STI transmission. We hypothesize that a) persons who use multiple substances over the course of the reference period (polysubstance use) will be more prone to CS than single-substance users and non-users; b) persons who use any type of substance right before or during sex are at a higher risk of CS than persons who do not use substances before/during sex; and c) the degree of increased risk of CS in persons who use substances (PWUS) will vary depending on substance type and demographic factors (age, gender, race).

## Methods

### Seek, test, treat, and retain (STTR) data collection and harmonization initiative

STTR was initiated by the National Institute on Drug Abuse and combines data from observational studies and trials intended to improve outcomes along the HIV care continuum for people involved in the criminal justice system and other vulnerable populations, many of whom have substance use disorders [36, 37]. Four US-based STTR studies contributed data to these analyses in order to enhance demographic, clinical, and geographic diversity. These were Brooklyn Community Action Project (BCAP, Brooklyn, NY); Baltimore-Rhode Island Get HIV Tested (BRIGHT, Baltimore, MD and Providence, RI); Seek, Test, and Retain (STAR, New York, NY); and Seek, Test, and Treat Strategies (STTS, Milwaukee County, WI). BRIGHT and STTS consisted of populations involved with the criminal justice system (probationers, parolees, and recent detainees about to be released); BCAP and STAR consisted of populations at high risk of HIV acquisition due to lifestyle factors (Table 1). We selected studies from the STTR consortium based on availability of illicit drug and alcohol use data, hereinafter referred to as substance use, and sexual risk behavior data. All participants ≥18 years of age who reported sexual activity were eligible for inclusion. Data were collected between 12/2011 and 06/2015.

**Table 1:** STTR studies

Study	Geographic Location	Population	Recent substance use	Substance use before/during sex	Reference Period	Participants Contributing Data
BCAP	Brooklyn, NY	Heterosexual individuals at high risk for HIV infection	Yes	Yes	30 days	2133 (31.5)
BRIGHT	Baltimore City, MD & Providence, RI	Probationers/ parolees	Yes	No	90 days	1537 (22.7)
STAR	New York, NY	African American substance-using men at risk for STI/HIV	Yes	Yes	30 days	768 (11.3)
STTS	Milwaukee County, WI	Detainees in the Wisconsin prison system detention center	Yes	No	90 days	2343 (34.6)
**TOTAL**						**6781**

*Abbreviations*: *BCAP* Brooklyn Community Action Project, *BRIGHT* Baltimore-Rhode Island Get HIV Tested, *STAR* Seek, Test, and Retain, *STTR* Seek, Test, and Treat Strategies

### Recruitment and sampling

BCAP and STAR used respondent-driven incentivized seed-based recruitment. BRIGHT and STTS used convenience sampling, with BRIGHT recruiting via flyers and posters within a community corrections office, and STTS by uniformly approaching all new detainees to invite participation. Data were collected either via structured self-administered computerized interview (BCAP, STTS) or by trained research interviewers (BRIGHT, STAR).

### Data sources

The STTR data repository integrates data from STTR studies, including clinical data such as standardized HIV-related information, as well as demographic, HIV transmission risk factors, and SU data obtained from enrollment interviews. All SU and CS variables focused on recent activity defined as the prior 1–3 months (timeframes were either 30 or 90 days). For studies including incarcerated participants, only non-incarcerated timeframes prior to incarceration were included. All studies used a variation of the AUDIT for alcohol use, with the exception of BCAP, which asked a single item querying frequency of alcohol use in the past 30 days. For drug use, all studies used variations of the ASSIST appropriate for individual study goals and population needs. SU was parameterized as a binary exposure (yes/no) and was defined as use of any of the following substances: binge alcohol (defined as ≥5 drinks per day for men and ≥ 4 for women), illicit opioids, cocaine/crack, methamphetamines/ stimulants, and other substances (e.g., hallucinogens, inhalants, barbiturates, synthetic drugs). An additional parameterization of illicit SU was also evaluated, which included the same drugs as above with the exception of binge alcohol use and cannabis (any alcohol use and/or cannabis use were included in the referent group). Participants were also asked about the timing of SU (before/during), sexual activity, and condom use over the recall period. Two overall SU exposure variables were created for analysis: 1) Any SU during the reference period, hereinafter referred to as recent substance use, and more specifically, 2) SU before or during sex. Main analyses did not include cannabis as our preliminary findings showed no association between cannabis and CS. We did, however, conduct sensitivity analyses including cannabis as an exposure. Each participant was categorized as either a non-user, a single-substance user, or a polysubstance user. SU before/during sex was included as a binary exposure (yes/no) from two of the included studies (Table 1). The outcome, engagement in any CS during the recall period, was harmonized across studies as a binary variable (yes/no).

### Statistical analyses

All statistical analyses was performed in Stata version 14 [38].

### Recent substance use

We examined demographic characteristics (including age, gender, race/ethnicity, sexual orientation, and education) and HIV risk factors both overall and by SU category (Table 2). We used individual-study, multivariable relative risk regression with robust confidence intervals to examine the relationship between CS among single- and poly-substance users compared to non-users [39]. Final models included age, race/ethnicity, and gender. Pooled effect estimates were calculated using traditional fixed-effects meta-analysis because we did not believe naïve pooling was appropriate given the heterogeneity of the study populations [40]. Additional analyses stratified by type of sex (vaginal sex only or vaginal and anal sex, from all 4 studies) and by partner self-reported HIV status (HIV-negative partners only, HIV-status unknown partners only, or HIV-negative and HIV-status unknown partners; data available from BRIGHT and STAR only). We assessed interaction between type of sex and substance use category as well as partner(s)’ self-reported HIV status and substance use category, however, we were not specifically powered to detect statistical interaction. Sensitivity analyses including cannabis as a substance and by recall period (30 or 90 days) were also conducted.

**Table 2:** Demographic characteristics by substance use category – Recent substance use

			Substance Use Category		
		Total	None	Single-Substance	Polysubstance
**N**	N	6781	3292 (48.6)	2222 (32.8)	1267 (18.7)
**Male**	6781	5573 (82.2)	2623 (79.7)	1814 (81.6)	1136 (89.7)
**Age**	6781	33 (26–45)	32 (25–44)	34 (26–45)	37 (27–47)
**Race/Ethnicity**	6781				
Black or African American		4073 (60.1)	2246 (68.2)	1280 (57.6)	547 (43.2)
White		748 (11.0)	252 (7.7)	209 (9.4)	287 (22.7)
Hispanic or Latino		1588 (23.4)	620 (18.8)	619 (27.9)	349 (27.6)
Other race		111 (1.6)	52 (1.6)	34 (1.5)	25 (2.0)
Two or more races		261 (3.9)	122 (3.7)	80 (3.6)	59 (4.7)
**Sexual Orientation**^**b**^	5244				
Heterosexual/Straight		4289 (81.8)	2191 (90.1)	1363 (77.8)	735 (69.3)
Homosexual/Gay/Lesbian/Queer/“Down-Lo”		62 (1.2)	13 (0.5)	22 (1.3)	27 (2.6)
Bisexual/Other		868 (16.6)	219 (9.0)	356 (20.3)	293 (27.6)
Refused/Missing		25 (0.5)	8 (0.3)	12 (0.7)	5 (0.5)
**Education**	6781				
High school or less		5270 (77.7)	2587 (78.6)	1710 (77.0)	973 (76.8)
Some college		1351 (19.9)	635 (19.3)	456 (20.5)	260 (20.5)
College graduate or above		157 (2.3)	67 (2.0)	56 (2.5)	34 (2.7)
Refused/Missing		3 (< 0.1)	3 (0.1)	0	0
**Risk Behaviors**^**c**^	6781				
*Number of sex partners*		1 (1–3)	1 (1–2)	2 (1–3)	2 (1–4)
*Unprotected sex*		5435 (80.2)	2528 (76.8)	1845 (83.0)	1062 (83.8)
*Injection Drug Use*					
Ever IDU		976 (14.4)	280 (8.5)	231 (10.4)	465 (36.7)
Recent IDU		355 (5.2)	–	64 (2.9)	291 (23.0)
**Substance Use**^**c**^	6781				
Alcohol		4142 (61.1)	1116 (33.9)	1946 (87.6)	1080 (85.2)
Binge alcohol		2734 (40.3)	–	1787 (80.4)	947 (74.7)
Cocaine/crack		907 (13.4)	–	107 (4.8)	800 (63.1)
Illicit opioids		917 (13.5)	–	178 (8.0)	739 (58.3)
Methamphetamine/stimulants		361 (5.3)	–	47 (2.1)	314 (24.8)
Cannabis		2370 (35.0)	734 (22.3)	896 (40.3)	740 (58.4)
Other		628 (9.3)	–	103 (4.6)	525 (41.4)

Data presented as median (IQR) or n (%) - percent may not sum to 100 due to rounding

^a^ None included participants who reported no binge alcohol use or illicit drug use (non-binge alcohol use and cannabis use allowed)

^b^ Sexual orientation was not collected in one study (BRIGHT)

^c^ Reference period: past 30 or 90 days (see Table 1)

### Substance use before/during sex

We examined demographic and clinical characteristics by SU before/during sex. We used individual-study, multivariable relative risk regression with robust confidence intervals to examine the relationship between CS among PWUS before/during sex compared to those who did not. Final models included age, race/ethnicity, and gender. Again, pooled effect estimates were calculated using traditional fixed-effects meta-analysis [40]. Additional analyses of CS among PWUS before/during sex compared to those who did not were performed after stratifying by type of sex. We assessed interaction between type of sex and substance use before and during sex as well, with the same limitation as previously mentioned.

### Time frame sensitivity analysis

We performed a time-frame sensitivity analysis to assess the potential effect of heterogenous recall periods.

## Results

### Recent substance use

Of the 6781 sexually active participants in the pooled analyses, median age of participants was 33 years (interquartile range (IQR): 26–45), 82% were male, and the majority had a high school education or less (78%). Approximately half (52%) of the included participants reported substance use, with 19% reporting polysubstance use during the reference period (Table 2, see Supplement Table 1 for demographic differences broken down by study). PWUS differed on some demographic characteristics from non-users. Single- and poly-substance users, compared to non-users, reported more sexual partners on average and more often identified as something other than heterosexual, despite reporting heterosexual sex. Polysubstance users, compared to both non-users and single-substance users, were more likely to be male (90% vs. 80 and 82%, respectively), older (37 years vs. 32 and 34 years, respectively), and white (23% vs. 8 and 9%, respectively). The most commonly reported substances among single-substance users were binge alcohol (80%), followed by illicit opioids (8%), cocaine/crack (5%), and other substances (5%). The most commonly reported substances among polysubstance users were binge alcohol (75%), followed by cocaine/crack (63%), illicit opioids (58%), and other substances (41%).

In regression analyses adjusted for demographic variables, PWUS were significantly more likely to engage in CS compared to non-users. Single-substance users were 8% more likely (95% confidence interval (CI): 5–11%, *p* < 0.001) to report recent CS and polysubstance users 9% more likely (95% CI: 5–13%, p < 0.001) to engage in this sexual risk behavior. A comparison of polysubstance users to single-substance users found no significant difference in likelihood of engaging in CS (RR = 1.02, 95% CI: 0.99–1.05, *p* = 0.26) (Table 3). There was no significant interaction by age and SU (results not shown). We also performed this analysis with only illicit substance use as the exposure (binge alcohol use moved to referent group), and inference did not change (see Table 3). As individual substances, binge alcohol, cocaine/crack, and illicit opioid use were each associated with CS [*p* = .04 (95% CI:1.00–1.07), *p* = .02 (95% CI:1.01–1.08), and *p* = .006 (95% CI:1.02–1.09), respectively] (see Supplement Table 2).

**Table 3:** Recent substance use and condomless sex

Recent substance use	RR	95% CI	***p***-value
**Overall**			
None^a^	Ref	–	–
Single substance	**1.08**	**1.05–1.11**	**< 0.001**
Polysubstance	**1.09**	**1.05–1.13**	**< 0.001**
Single- vs. poly-substance	1.02	0.99–1.05	0.26
**Overall – Illicit substance use only**			
None^b^	Ref	–	–
Single substance	**1.06**	**1.02–1.09**	**0.002**
Polysubstance	**1.09**	**1.05–1.13**	**< 0.001**
Single- vs. poly-substance	1.01	0.97–1.05	0.68
**By type of sex**			
Vaginal sex only	Ref	–	–
Vaginal and anal sex	**1.21**	**1.16–1.26**	**< 0.001**
*Vaginal sex only*			
None^a^	Ref	–	–
Single substance	**1.09**	**1.05–1.14**	**< 0.001**
Polysubstance	**1.10**	**1.05–1.15**	**< 0.001**
Single- vs. poly-substance	1.01	0.96–1.05	0.81
*Vaginal and anal sex*			
None^a^	Ref	–	–
Single substance	**1.05**	**1.02–1.09**	**0.004**
Polysubstance	**1.08**	**1.04–1.12**	**< 0.001**
Single- vs. poly-substance	1.03	0.99–1.06	0.12
**By HIV status of partner**			
HIV-negative partners only	Ref	–	–
HIV-unknown partners only	**0.92**	**0.86–0.98**	**0.01**
HIV-negative & HIV-unknown partners only	**1.11**	**1.04–1.19**	**0.001**
*HIV-negative partners only*			
None^a^	Ref		
Single substance	**1.08**	**1.02–1.13**	**0.004**
Polysubstance	**1.07**	**1.01–1.14**	**0.02**
Single- vs. poly-substance	0.99	0.94–1.05	0.81
*HIV-unknown partners only*			
None^a^	Ref	–	–
Single substance	1.04	0.95–1.13	0.39
Polysubstance	0.97	0.88–1.07	0.57
Single- vs. poly-substance	0.94	0.85–1.03	0.20
*HIV-negative & HIV-unknown partners only*			
None^a^	Ref	–	–
Single substance	**1.09**	**1.01–1.18**	**0.03**
Polysubstance	**1.13**	**1.06–1.20**	**< 0.001**
Single- vs. poly-substance	1.04	0.98–1.10	0.22

*Abbreviations*: *CI* Confidence interval, *RR* Relative risk

Models adjusted for age, race/ethnicity, and gender

^a^ None included participants who reported no binge alcohol use or illicit drug use (non-binge alcohol use and cannabis use allowed)

^b^ None included participants who reported no illicit drug use (any alcohol use and cannabis use allowed)

Type of sex was associated with CS, with participants reporting vaginal and anal sex 21% more likely (95% CI: 16–26%) to engage in CS compared to those reporting vaginal sex only (*p* < 0.001). Among participants having only vaginal sex, use of any substances was associated with a higher likelihood of CS compared to non-users, with single-substance users 9% more likely (95% CI: 5–14%, *p* < 0.001) and polysubstance users 10% more likely (95% CI: 5–15%, *p* < 0.001) to report CS. Among participants having both vaginal and anal sex, substance use also increased the likelihood of CS compared to non-users, with single-substance users 5% more likely (95% CI: 2–9%, *p* = 0.004) and polysubstance users 8% more likely (95% CI: 4–12%, *p* < 0.001) to report CS (Table 3). There was no significant interaction between type of sex and substance use category (results not shown).

Partner(s’) self-reported HIV status was also associated with CS, with an 8% lower likelihood (95% CI: 2–14%, *p* = 0.01) of CS among participants reporting HIV-status unknown partners only compared to exclusively HIV-negative partners and a 11% higher likelihood (95% CI: 4–19%, *p* = 0.001) of CS among participants reporting both HIV-negative and HIV-status unknown partners compared to exclusively HIV-negative partners. Substance use was significantly associated with higher likelihood of CS among participants reporting exclusively HIV-negative partners and participants reporting both HIV-negative and HIV-status unknown partners, but not among participants reporting HIV-status unknown partners only. Use of any substances increased the likelihood of CS by 8% (95% CI: 2–13%, *p* = 0.004) for single-substance users and 7% (95% CI: 1–14%, *p* = 0.02) for polysubstance users compared to non-users among participants reporting exclusively HIV-negative partners. Use of any substances increased the likelihood of CS by 9% (95% CI: 1–18%, *p* = 0.03) for single-substance users and 13% (95% CI: 6–20%, *p* < 0.001) for polysubstance users compared to non-users among participants reporting HIV-negative and HIV-status unknown partners (Table 3). There was no significant interaction between partner(s)’ HIV status and substance use category (results not shown).

For individual study estimates and meta-analytic forest plots for all associations presented for recent substance use, see Supplemental Figures 1, 2, 3, 4, 5, 6, 7, 8 and 9.

### Substance use before or during sex

Among participants (*n* = 2915) that had information on substance use before/during sex and CS during the reference period, median age was 40 (IQR: 28–49), 69% male, and most had high school education or less (77%). Just over half (52%) of the participants reported substance use before/during sex (Table 4, see Supplement Table 3 for demographics broken down by study). Similar to what we observed with recent PWUS, participants who used substances before/during sex reported more sexual partners on average and despite reporting heterosexual behavior more often identified as something other than heterosexual. Additionally, those who used substances before/during sex reported higher rates of use across all substance categories, had higher rates of injection drug use (6% vs. 1%), and tended to be male (84% vs. 54%) and Hispanic or Latino (47% vs. 33%). The most commonly reported substances used before/during sex were binge alcohol (65%), polysubstance use (57%), cocaine/crack (21%), and illicit opioids (18%).

**Table 4:** Demographic characteristics among those using substances before or during sex

			Substance use before/during sex	
		Total	No	Yes
**N**	N	2915	1397 (47.9)	1518 (52.1)
**Male**	2915	2021 (69.3)	758 (54.3)	1263 (83.2)
**Age**	2915	40 (28–49)	37 (26–48)	41 (29–49)
**Race/Ethnicity**	2915			
Black or African American		1701 (58.4)	912 (65.3)	789 (52.0)
White		0	0	0
Hispanic or Latino (alone or in combination with race)		1169 (40.1)	460 (32.9)	709 (46.7)
Two or more races		45 (1.5)	25 (1.8)	20 (1.3)
**Sexual Orientation**	2915			
Heterosexual/Straight		2002 (68.7)	1168 (83.6)	834 (54.9)
Homosexual/Gay/Lesbian/Queer/Down-Lo		55 (1.9)	13 (0.9)	42 (2.8)
Bisexual/Other		843 (28.9)	212 (15.2)	631 (41.6)
Refused/DK/Missing		15 (0.5)	4 (0.3)	11 (0.7)
**Education**	2915			
High school or less		2251 (77.2)	1084 (77.6)	1167 (76.9)
Some college		572 (19.6)	278 (19.9)	294 (19.4)
College graduate or above		91 (3.1)	34 (2.4)	57 (3.8)
Refused/DK/Missing		1 (< 0.1)	1 (0.1)	0
**Risk Behaviors**^**a**^	2915			
*Number of sex partners*		2 (1–3)	1 (1–2)	2 (1–3)
*Unprotected sex*		2425 (83.2)	1110 (79.5)	1315 (86.6)
*Injection Drug Use*				
Ever IDU		371 (12.7)	116 (8.3)	255 (16.8)
Recent IDU		92 (3.2)	8 (0.6)	84 (5.5)
**Substance Use**^**a**^	2915			
Alcohol		2133 (73.2)	812 (58.1)	1321 (87.0)
Binge alcohol		1419 (48.7)	437 (31.3)	982 (64.7)
Cocaine/crack		334 (11.5)	19 (1.4)	315 (20.8)
Illicit opioids		285 (9.8)	17 (1.2)	268 (17.7)
Methamphetamine/stimulants		73 (2.5)	10 (0.7)	63 (4.2)
Cannabis		899 (30.8)	192 (13.7)	707 (46.6)
Other		180 (6.2)	19 (1.4)	161 (10.6)
Polysubstance		1039 (35.6)	175 (12.5)	864 (56.9)

Data presented as median (IQR) or n (%) – percent may not sum to 100 due to rounding

^a^ Reference period: past 30 days

In regression analyses adjusted for demographic variables, substance use before/during sex was significantly associated with CS, with a 16% increased likelihood (95% CI: 12–20%, *p* < 0.001) of CS compared to participants that did not use substances during sex (Table 5). The association between substance use during/before sex and CS did not change across age levels (results not shown). There was no significant interaction by age and substance use before/during sex (results not shown).

**Table 5:** Substance use before or during sex and condomless sex

Substance use before/during sex	RR	95% CI	p-value
**Overall**			
No	Ref	–	–
Yes	**1.16**	**1.12–1.20**	**< 0.001**
**By type of sex**			
Vaginal sex only	Ref	–	–
Vaginal and anal sex	**1.18**	**1.11–1.26**	**< 0.001**
*Vaginal sex only*			
No	Ref	–	–
Yes	**1.20**	**1.12–1.29**	**< 0.001**
*Vaginal and anal sex*			
No	Ref	–	–
Yes	**1.07**	**1.03–1.11**	**0.002**

*Abbreviations*: *CI* Confidence interval, *RR* Relative risk

Models adjusted for age, race/ethnicity, and gender

Again, type of sex was associated with CS, with an 18% higher likelihood (95% CI: 11–26%, p < 0.001) of CS among participants reporting vaginal and anal sex compared to vaginal sex only. Among participants having only vaginal sex, consumption of one or more substances during sex increased the likelihood of CS by 20% (95% CI: 12–29%, p < 0.001). Among participants having both vaginal and anal sex, consumption of one or more substances during sex increased the likelihood of CS by 7% (95% CI: 3–11%, *p* = 0.002) (Table 5). There was no significant interaction between type of sex and substance use before/during sex (results not shown).

For individual study estimates and meta-analytic forest plots for all associations presented for substance use before/during sex, see Supplemental Figures 10, 11, 12 and 13.

### Time frame sensitivity analysis

While the 30-day recall period showed stronger associations, overall inference and patterns were similar (see Supplement Tables 4 and 5).

### Sensitivity analyses with Cannabis

Sensitivity analyses found that cannabis was not associated with CS when used in the absence of other substances (RR = 0.99, 95% CI: 0.94–1.04, *p* = 0.61) (Supplement Table 6). Additionally, even with increasing frequency of use, cannabis was not associated with engagement in CS (RR range = 1.00–1.05, *p*-value range: 0.24–0.92) (Supplement Table 7).

## Discussion

In this large, cross-sectional study with a diverse sample of substance-using HIV-uninfected adults reporting heterosexual sex, we note several key findings. The use of substances excluding cannabis, was associated with CS relative to non-substance use. This was true of both recent substance use (in either the previous 30 or 90 days), as well as substance use before/during sex. Recent polysubstance use also increased CS risk, though not more so than recent single-substance use. The lack of excess risk among polysubstance compared to single-substance users does not align with findings from a large study of HIV-uninfected MSM [34]. Before or during sex, any substance use, with the exception of cannabis, increased CS. Notably, age did not modify the effect between SU and increased risk of CS. The finding that most substances increase CS contrasts from that of a recent systemic review of substance administration studies, which found alcohol to be independently associated with sexual risk behavior, that cocaine increased it, and cannabis decreased it [41].

A key finding is the strong correlation between concurrent sexual and drug use risk. Previous studies using a variety of methods inconsistently show a temporal relationship between concurrent drug/alcohol use and sexual risk [42], likely due to pitfalls and differences in collection methods. Timeline follow-back, with 14- or 30-day recall, is fraught with recall bias [43]. Daily diaries are constrained because entries are not made in near real-time and do not measure intentions before events occur [44]. Newer event-level methods, like ecological momentary assessment [45, 46], may further disentangle this association.

Cannabis use did not increase the risk of CS, neither in the context of recent use nor before/during sex. Frequency of cannabis use did not change CS risk. While a similar lack of effect on CS and STI outcomes has been apparent in studies of adolescents, young adults, and MSM [42, 47–52],, other studies have found the opposite [27–30]. Several wide-ranging factors may be driving differences in findings; these include population-level factors (e.g., age, gender, sexual orientation); social factors (e.g., relationship type and duration, power dynamics surrounding sexual behavior, and the role of intentionality to use condoms in the context of cannabis use); factors specific to cannabis and its use itself (e.g., drug potency, dependency, tolerance to its effects); study design factors, such as social desirability bias from particular data collection methods. More research, ideally a meta-analytic review, or a large representative sample of the whole source population with careful sub-group testing is needed to help reconcile discrepancies between findings.

Due to mixed findings of a correlation between cannabis use and CS in the wider literature, findings here are restricted to an older population than previously reported. Our relatively mature adult cohort, many of whom represent highly marginalized populations and have long histories of cannabis and other substance use, may have developed a tolerance to the effects of cannabis. Frequent cannabis users have shown an increased tolerance to its impairing effects [53], even when also intoxicated by alcohol [54]; members of our cohort may have developed an ability to function under its influence or under that of multiple combined substances. Results may differ among less-experienced users such as youth/adolescents, for whom the effects of cannabis have been found to be particularly detrimental to brain function [55, 56]; indeed, a meta-analysis of event-based studies found cannabis use around the time of intercourse increased the odds of CS among adolescents, but not adults [57]. Due to the ambiguity of cannabis’s role in CS across populations relative to other non-cannabis substances, we recommend that future analyses of poly-substance impact on CS analyze cannabis use independently from other grouped substances in order to avoid potentially underestimating the impact of non-cannabis substances.

We found that having exclusively HIV-negative partner/s increased the likelihood of CS. Having exclusively HIV-status unknown partners did not, even in the context of substance use before/during sex. Assuming that not knowing a partner’s HIV status implies less overall familiarity with the partner, a possible explanation for this finding is that lower partner familiarity is associated with increased condom use, perhaps indicative of greater concern or consciousness for potential HIV/STI transmission risk and/or pregnancy risk. Conversely, the higher rate of CS observed among those with exclusively HIV-negative partners may in part reflect practices within long-term and/or perceivably monogamous partnerships in which STI/HIV transmission is thought to be of low concern, diminishing the felt need to use condoms. Among those with a mix of HIV-status unknown and HIV-negative partners, CS was also higher. However, with this group, we lacked data regarding with which partners (HIV-negative or HIV-status unknown) condoms were and were not used. Our findings among those with partner/s of exclusive serostatus (all negative or all unknown) suggest that the high rate of CS among those with both types of partners is driven primarily by the low condom use observed between HIV-negative partners.

Based on these findings, it may be advisable for HIV/STI prevention efforts as well as care providers to assess HIV/STI transmission risk among those with exclusively HIV-negative partners, particularly non-cannabis using PWUS, and to assess HIV/STI transmission risk and prevention practices among those reporting any partners for whom HIV/STI status is unknown. Of concern, we found that CS was more likely to occur among those having anal sex in addition to vaginal sex, compared to those reporting exclusively vaginal sex; substance use increased likelihood of CS for both of these groups. This has particularly strong implications for HIV transmission risk, given the higher risk of infection known to occur in anal compared to vaginal sex, underscoring the need to identify such risk behaviors, including recent substance use, at point-of-care. It is also essential that gender dynamics and sexual power be considered with sensitive instrumentation and interventions for those who might report lack of personal agency for protection, so that interventions could address these differentials and help empower participants with more at-risk behaviors. Finally, we note that participant age did not have an effect on likelihood of CS, neither for recent PWUS nor those who used before/during sex, indicating a need to address sexual risk behavior across the life span.

### Strengths

A strength of our study is its sample size and the demographic and geographic diversity of participants.

### Limitations

We note that study sites did not use uniform time frames in their measures. Additionally, because different studies used different instruments, collection of covariates differed by study and data were not necessarily available for all potential confounders. Because the data were cross-sectional, only associations and not causation could be determined. While we used CS as our outcome, we cannot infer any increased level of developing a STI. We note that because nuanced data was not available on partner serosorting, condom use behavior lacked context. We also note the possibility self-report bias in the areas of substance use, sexual behavior, and HIV negative serostatus. In addition, this paper focuses on condomless sex. However, clearly there are other approaches to address in order to reduce HIV/STI transmission, including the use of pre-exposure prophylaxis and needle exchange programs for injection drug use.

## Conclusion

With the exception of cannabis, any recent substance use was associated with an increased likelihood of engaging in CS, whether used in the past 30–90 days, or before/during sex. Polysubstance use, however, relative to single substance use, did not further elevate this risk. Higher frequency of cannabis use did not increase the risk of CS. Future studies of the impact of substance use on behavior should consider analyzing cannabis use independently from other substances. For non-cannabis-using PWUS, CS was higher for participants with exclusively HIV-negative partners and for those reporting both anal and vaginal sex in the past 30 or 90 days. Conversely, participants reporting exclusively HIV-status unknown partners in the same reference period did not have a higher risk of CS. STI prevention efforts among non-cannabis-using PWUS should not overlook STI risk assessment for those reporting HIV-negative partners.

## Supplementary Information

**Additional file 1.**

## Data Availability

Data and materials are archived by both the DCC and NIDA. They can be made available upon reasonable request, with a concept proposal and fully executed data use agreement (due to the sensitivity of the data). Interested investigators can email jacd@uw.edu for more details.
