# Supplementary material for: Individual and poly-substance use and condomless sex among HIV-uninfected adults reporting heterosexual sex in a multi-site cohort
Source: BMC Public Health. 2021 Nov 4;21:2002. doi: 10.1186/s12889-021-12026-7 (PMC8567631; doi:10.1186/s12889-021-12026-7)

**Supplemental Table 1. Demographic characteristics by Study – Recent substance use**

|  |  |  | **Studies** | | | |
| --- | --- | --- | --- | --- | --- | --- |
|  |  | **Total** | **BCAP** | **BRIGHT** | **STAR** | **STTS** |
| **N** | N | 6,781 | 2,133 | 1,537 | 768 | 2,343 |
| **Male** | 6,781 | 5,573 (82.2) | 1,243 (58.3) | 1,285 (83.6) | 768 (100) | 2,277 (97.2) |
| **Age** | 6,781 | 33 (26-45) | 36 (26-48) | 37 (27-46) | 44 (32-50) | 29 (24-35) |
| **Race/Ethnicity** | 6,781 |  |  |  |  |  |
| Black or African American |  | 4,073 (60.1) | 1,503 (70.5) | 877 (57.1) | 191 (24.9) | 1,502 (64.1) |
| White |  | 748 (11.0) | 0 | 345 (22.5) | 0 | 403 (17.2) |
| Hispanic or Latino |  | 1,588 (23.4) | 588 (27.6) | 200 (13.0) | 574 (74.7) | 226 (9.7) |
| Other race |  | 111 (1.6) | 0 | 55 (3.6) | 0 | 56 (2.4) |
| Two or more races |  | 261 (3.9) | 42 (2.0) | 60 (3.9) | 3 (0.4) | 156 (6.7) |
| **Sexual Orientation^2^** | 5,244 |  |  |  |  |  |
| Heterosexual/Straight |  | 4,289 (81.8) | 1,893 (88.8) | -- | 116 (15.1) | 2,280 (97.3) |
| Homosexual/Gay/Lesbian/  Queer/”Down-Lo” |  | 62 (1.2) | 15 (0.7) | -- | 39 (5.1) | 8 (0.3) |
| Bisexual/Other |  | 868 (16.6) | 223 (10.5) | -- | 601 (78.3) | 44 (1.9) |
| Refused/Missing |  | 25 (0.5) | 2 (0.1) | -- | 12 (1.6) | 11 (0.5) |
| **Education** | 6,781 |  |  |  |  |  |
| High school or less |  | 5,270 (77.7) | 1,647 (77.2) | 1,274 (82.9) | 595 (77.4) | 1,754 (74.9) |
| Some college |  | 1,351 (19.9) | 437 (20.5) | 240 (15.6) | 130 (16.9) | 544 (23.2) |
| College graduate or above |  | 157 (2.3) | 48 (2.3) | 23 (1.5) | 43 (5.6) | 43 (1.8) |
| Refused/Missing |  | 3 (<0.1) | 1 (0.1) | 0 | 0 | 2 (0.1) |
| **Risk Behaviors^3^** | 6,781 |  |  |  |  |  |
| *Number of sex partners* |  | 1 (1-3) | 1 (1-2) | 1 (1-2) | 3 (2-3) | 2 (1-3) |
| *Unprotected sex* |  | 5,435 (80.2) | 1,798 (84.3) | 1,188 (77.3) | 621 (80.9) | 1,828 (78.0) |
| *Injection Drug Use* |  |  |  |  |  |  |
| Ever IDU |  | 976 (14.4) | 194 (9.1) | 330 (21.5) | 181 (23.6) | 271 (11.6) |
| Recent IDU |  | 355 (5.2) | 41 (1.9) | 81 (5.3) | 54 (7.0) | 179 (7.6) |
| **Substance Use^3^** | 6,781 |  |  |  |  |  |
| Alcohol |  | 4,142 (61.1) | 1,478 (69.3) | 813 (52.9) | 634 (82.6) | 1,217 (51.9) |
| Binge alcohol |  | 2,734 (40.3) | 919 (43.1) | 491 (32.0) | 485 (63.2) | 839 (35.8) |
| Cocaine/crack |  | 907 (13.4) | 77 (3.6) | 177 (11.5) | 266 (34.6) | 387 (16.5) |
| Illicit opioids |  | 917 (13.5) | 85 (4.0) | 228 (14.8) | 210 (27.3) | 394 (16.8) |
| Methamphetamine/stimulants |  | 361 (5.3) | 43 (2.0) | 6 (0.4) | 30 (3.9) | 282 (12.0) |
| Cannabis |  | 2,370 (35.0) | 550 (25.8) | 466 (30.3) | 362 (47.1) | 992 (42.3) |
| Other |  | 628 (9.3) | 96 (4.5) | 50 (3.3) | 90 (11.7) | 392 (16.7) |
| Polysubstance |  | 2,625 (38.7) | 547 (25.6) | 524 (34.1) | 506 (65.9) | 1,048 (44.7) |

Data presented as median (IQR) or n (%) - percent may not sum to 100 due to rounding.

^1^ Reference period for number of sex partners, unprotected sex, injection drug use, and substance use: past 30 or 90 days.

**Supplemental Table 2. Recent substance use and condomless sex: Individual substances**

| **Recent substance use** | **RR** | **95% CI** | **p-value** |
| --- | --- | --- | --- |
| **Overall** |  |  |  |
| **Binge alcohol** | **1.03** | **1.00-1.07** | **0.04** |
| **Cocaine/crack** | **1.05** | **1.01-1.08** | **0.02** |
| **Illicit opioids** | **1.05** | **1.02-1.09** | **0.006** |

**Abbreviations: CI, confidence interval; RR, relative risk.**

**Models adjusted for age, race/ethnicity, and gender.**

**Supplemental Table 3. Demographic characteristics by Study – Substance Use Before or During Sex**

|  |  |  | **Studies** | |
| --- | --- | --- | --- | --- |
|  |  | **Total** | **BCAP** | **STAR** |
| **N** | N | 2,915 | 1,397(47.9) | 1,518 (52.1) |
| **Male** | 2,915 | 2,021 (69.3) | 1,229 (57.9) | 792 (100) |
| **Age** | 2,915 | 40 (28-49) | 36 (26-48) | 44 (32-51) |
| **Race/Ethnicity** | 2,915 |  |  |  |
| Black or African American |  | 1,701 (58.4) | 1,503 (70.8) | 198 (25.0) |
| White |  | 0 | 0 | 0 |
| Hispanic or Latino (alone or in  combination with race) |  | 1,169 (40.1) | 578 (27.2) | 591 (74.6) |
| Two or more races |  | 45 (1.5) | 42 (2.0) | 3 (0.4) |
| **Sexual Orientation** | 2,915 |  |  |  |
| Heterosexual/Straight |  | 2,002 (68.7) | 1,883 (88.7) | 119 (15.0) |
| Homosexual/Gay/Lesbian/  Queer/Down-Lo |  | 55 (1.9) | 15 (0.7) | 40 (5.1) |
| Bisexual/Other |  | 843 (28.9) | 223 (10.5) | 620 (78.3) |
| Refused/DK/Missing |  | 15 (0.5) | 2 (0.1) | 13 (1.6) |
| **Education** | 2,915 |  |  |  |
| High school or less |  | 2,251 (77.2) | 1,638 (77.2) | 613 (77.4) |
| Some college |  | 572 (19.6) | 437 (20.6) | 135 (17.1) |
| College graduate or above |  | 91 (3.1) | 47 (2.2) | 44 (5.6) |
| Refused/DK/Missing |  | 1 (<0.1) | 1 (0.1) | 0 |
| **Risk Behaviors^1^** | 2,915 |  |  |  |
| *Number of sex partners* |  | 2 (1-3) | 1 (1-2) | 2 (1-3) |
| *Unprotected sex* |  | 2,425 (83.2) | 1,787 (84.2) | 638 (80.6) |
| *Injection Drug Use* |  |  |  |  |
| Ever IDU |  | 371 (12.7) | 187 (8.8) | 184 (23.2) |
| Recent IDU |  | 92 (3.2) | 38 (1.8) | 54 (6.8) |
| **Substance Use^1^** | 2,915 |  |  |  |
| Alcohol |  | 2,133 (73.2) | 1,480 (69.7) | 653 (82.5) |
| Binge alcohol |  | 1,419 (48.7) | 918 (43.2) | 501 (63.3) |
| Cocaine/crack |  | 334 (11.5) | 67 (3.2) | 267 (33.7) |
| Illicit opioids |  | 285 (9.8) | 75 (3.5) | 210 (26.5) |
| Methamphetamine/stimulants |  | 73 (2.5) | 43 (2.0) | 30 (3.8) |
| Cannabis |  | 899 (30.8) | 537 (25.3) | 362 (45.7) |
| Other |  | 180 (6.2) | 90 (4.2) | 90 (11.4) |
| Polysubstance |  | 1,039 (35.6) | 532 (25.1) | 507 (64.0) |

Data presented as median (IQR) or n (%) - percent may not sum to 100 due to rounding.

^1^ Reference period for number of sex partners, unprotected sex, injection drug use, and substance use: past 30 days.

**Supplemental Table 4. Recent substance use and condomless sex: 30-day recall period**

| **Recent substance use** | **RR** | **95% CI** | **p-value** |
| --- | --- | --- | --- |
| **Overall** |  |  |  |
| **None ^1^** | **Ref** | **--** | **--** |
| **Single substance** | **1.09** | **1.05-1.13** | **<0.001** |
| **Polysubstance** | **1.14** | **1.08-1.20** | **<0.001** |
| **Single- vs. poly-substance** | **1.07** | **1.02-1.12** | **0.006** |
| **Overall – Illicit substance use only** |  |  |  |
| **None ^2^** | **Ref** | **--** | **--** |
| **Single substance** | **1.08** | **1.02-1.14** | **0.01** |
| **Polysubstance** | **1.15** | **1.10-1.20** | **<0.001** |
| **Single- vs. poly-substance** | **1.06** | **1.00-1.13** | **0.05** |
| **By type of sex** |  |  |  |
| **Vaginal sex only** | **Ref** | **--** | **--** |
| **Vaginal and anal sex** | **1.18** | **1.10-1.26** | **<0.001** |
| ***Vaginal sex only*** |  |  |  |
| **None ^1^** | **Ref** | **--** | **--** |
| **Single substance** | **1.12** | **1.04-1.21** | **0.002** |
| **Polysubstance** | **1.26** | **1.14-1.39** | **<0.001** |
| **Single- vs. poly-substance** | **1.12** | **1.02-1.23** | **0.02** |
| ***Vaginal and anal sex*** |  |  |  |
| **None ^1^** | **Ref** | **--** | **--** |
| **Single substance** | **1.06** | **1.01-1.10** | **0.01** |
| **Polysubstance** | **1.08** | **1.02-1.14** | **0.005** |
| **Single- vs. poly-substance** | **1.04** | **0.99-1.09** | **0.11** |

**Abbreviations: CI, confidence interval; RR, relative risk.**

**Models adjusted for age, race/ethnicity, and gender.**

**^1^ None included participants who reported no binge alcohol use or illicit drug use (non-binge alcohol use and cannabis use allowed).**

**^2^ None included participants who reported no illicit drug use (any alcohol use and cannabis use allowed).**

**Supplemental Table 5. Recent substance use and condomless sex: 90-day recall period**

| **Recent substance use** | **RR** | **95% CI** | **p-value** |
| --- | --- | --- | --- |
| **Overall** |  |  |  |
| **None ^1^** | **Ref** | **--** | **--** |
| **Single substance** | **1.07** | **1.03-1.11** | **<0.001** |
| **Polysubstance** | **1.05** | **1.01-1.10** | **0.02** |
| **Single- vs. poly-substance** | **0.98** | **0.93-1.02** | **0.29** |
| **Overall – Illicit substance use only** |  |  |  |
| **None ^2^** | **Ref** | **--** | **--** |
| **Single substance** | **1.05** | **1.00-1.09** | **0.04** |
| **Polysubstance** | **1.02** | **0.96-1.07** | **0.58** |
| **Single- vs. poly-substance** | **0.97** | **0.91-1.02** | **0.22** |
| **By type of sex** |  |  |  |
| **Vaginal sex only** | **Ref** | **--** | **--** |
| **Vaginal and anal sex** | **1.23** | **1.17-1.30** | **<0.001** |
| ***Vaginal sex only*** |  |  |  |
| **None ^1^** | **Ref** | **--** | **--** |
| **Single substance** | **1.08** | **1.03-1.13** | **0.001** |
| **Polysubstance** | **1.06** | **1.00-1.11** | **0.04** |
| **Single- vs. poly-substance** | **0.97** | **0.92-1.03** | **0.30** |
| ***Vaginal and anal sex*** |  |  |  |
| **None ^1^** | **Ref** | **--** | **--** |
| **Single substance** | **1.05** | **0.98-1.11** | **0.14** |
| **Polysubstance** | **1.07** | **1.01-1.14** | **0.02** |
| **Single- vs. poly-substance** | **1.01** | **0.97-1.06** | **0.55** |
| **By HIV status of partner** |  |  |  |
| **HIV-negative partners only** | **Ref** | **--** | **--** |
| **HIV-unknown partners only** | **0.92** | **0.86-0.98** | **0.01** |
| **HIV-negative & HIV-unknown partners only** | **1.11** | **1.04-1.19** | **0.001** |
| ***HIV-negative partners only*** |  |  |  |
| **None ^1^** | **Ref** |  |  |
| **Single substance** | **1.08** | **1.02-1.13** | **0.004** |
| **Polysubstance** | **1.07** | **1.01-1.14** | **0.02** |
| **Single- vs. poly-substance** | **0.99** | **0.94-1.05** | **0.81** |
| ***HIV-unknown partners only*** |  |  |  |
| **None ^1^** | **Ref** | **--** | **--** |
| **Single substance** | **1.04** | **0.95-1.13** | **0.39** |
| **Polysubstance** | **0.97** | **0.88-1.07** | **0.57** |
| **Single- vs. poly-substance** | **0.94** | **0.85-1.03** | **0.20** |
| ***HIV-negative & HIV-unknown partners only*** |  |  |  |
| **None ^1^** | **Ref** | **--** | **--** |
| **Single substance** | **1.09** | **1.01-1.18** | **0.03** |
| **Polysubstance** | **1.13** | **1.06-1.20** | **<0.001** |
| **Single- vs. poly-substance** | **1.04** | **0.98-1.10** | **0.22** |

**Abbreviations: CI, confidence interval; RR, relative risk.**

**Models adjusted for age, race/ethnicity, and gender.**

**^1^ None included participants who reported no binge alcohol use or illicit drug use (non-binge alcohol use and cannabis use allowed).**

**^2^ None included participants who reported no illicit drug use (any alcohol use and cannabis use allowed).**

**Supplemental Table 6. Recent cannabis use and/or recent binge alcohol use and condomless sex**

| **Recent substance use** | **RR** | **95% CI** | **p-value** |
| --- | --- | --- | --- |
| None^1^ | Ref | -- | -- |
| Binge alcohol only | **1.06** | **1.02-1.09** | **0.002** |
| Cannabis only | 0.99 | 0.94-1.04 | 0.61 |
| Only binge alcohol and cannabis | **1.09** | **1.05-1.13** | **<0.001** |

Abbreviations: CI, confidence interval; RR, relative risk.

Models adjusted for age, race/ethnicity, and gender.

^1^ None included participants who reported no binge alcohol use or illicit drug use (non-binge alcohol use and cannabis use allowed).

**Supplemental Table 7. Recent cannabis use frequency and condomless sex**

| **Recent substance use** | **RR** | **95% CI** | **p-value** |
| --- | --- | --- | --- |
| **Cannabis use frequency^1^** |  |  |  |
| Only a few times | Ref | -- | -- |
| 1-3 times per month | 1.04 | 0.97, 1.12 | 0.27 |
| About once a week | 1.05 | 0.96, 1.14 | 0.27 |
| 2-5 times per week | 1.00 | 0.93, 1.07 | 0.92 |
| About once a day | 1.03 | 0.98, 1.09 | 0.24 |
| Only a few times | Ref | -- | -- |

Abbreviations: CI, confidence interval; CS, condomless sex; RR, relative risk.

Models adjusted for age, race/ethnicity, and gender.

^1^ Cannabis users includes participants that reported any cannabis, whether alone or in conjunction with other substance.

**Supplemental Figure 1**


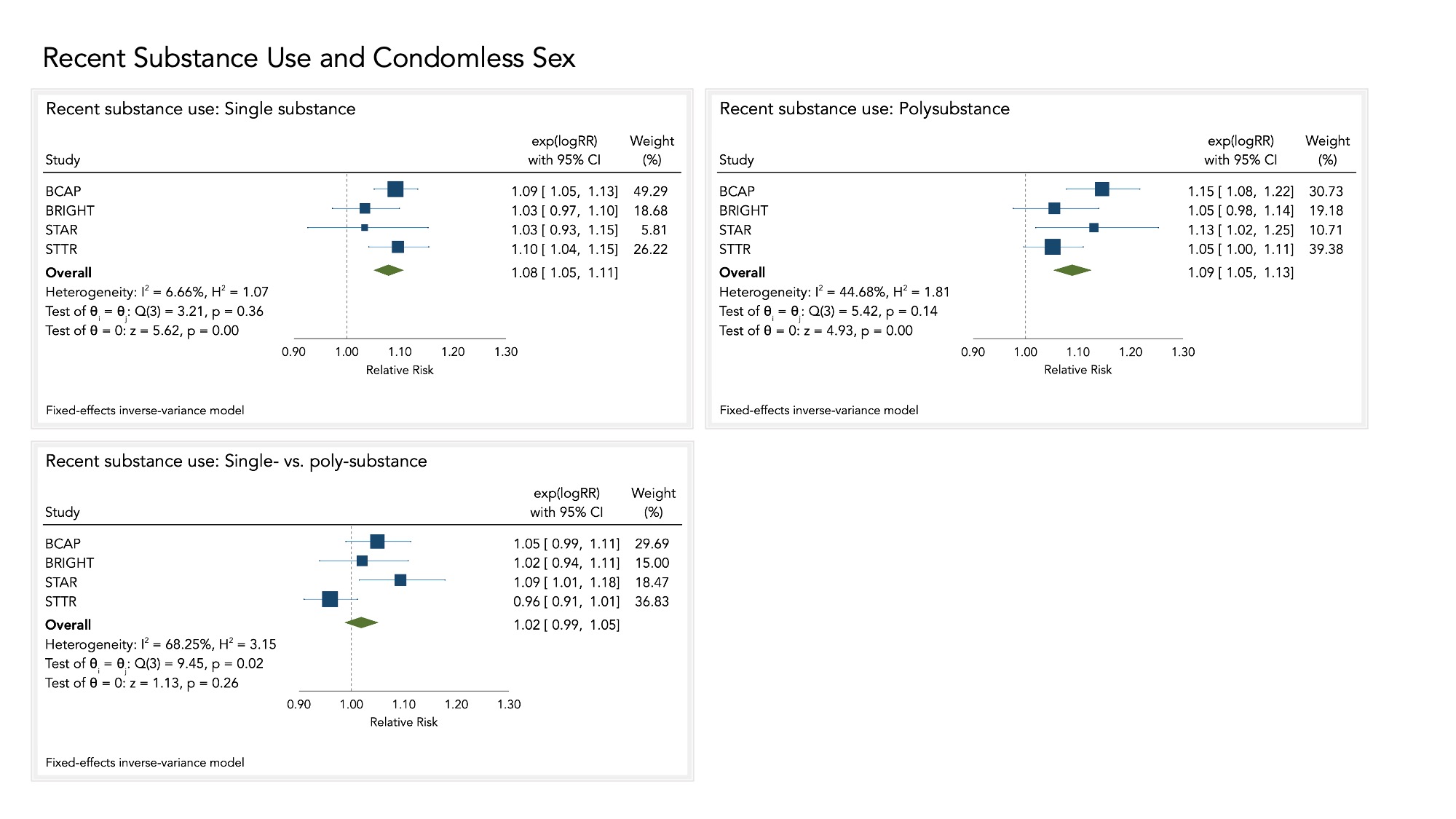


**Supplemental Figure 2**


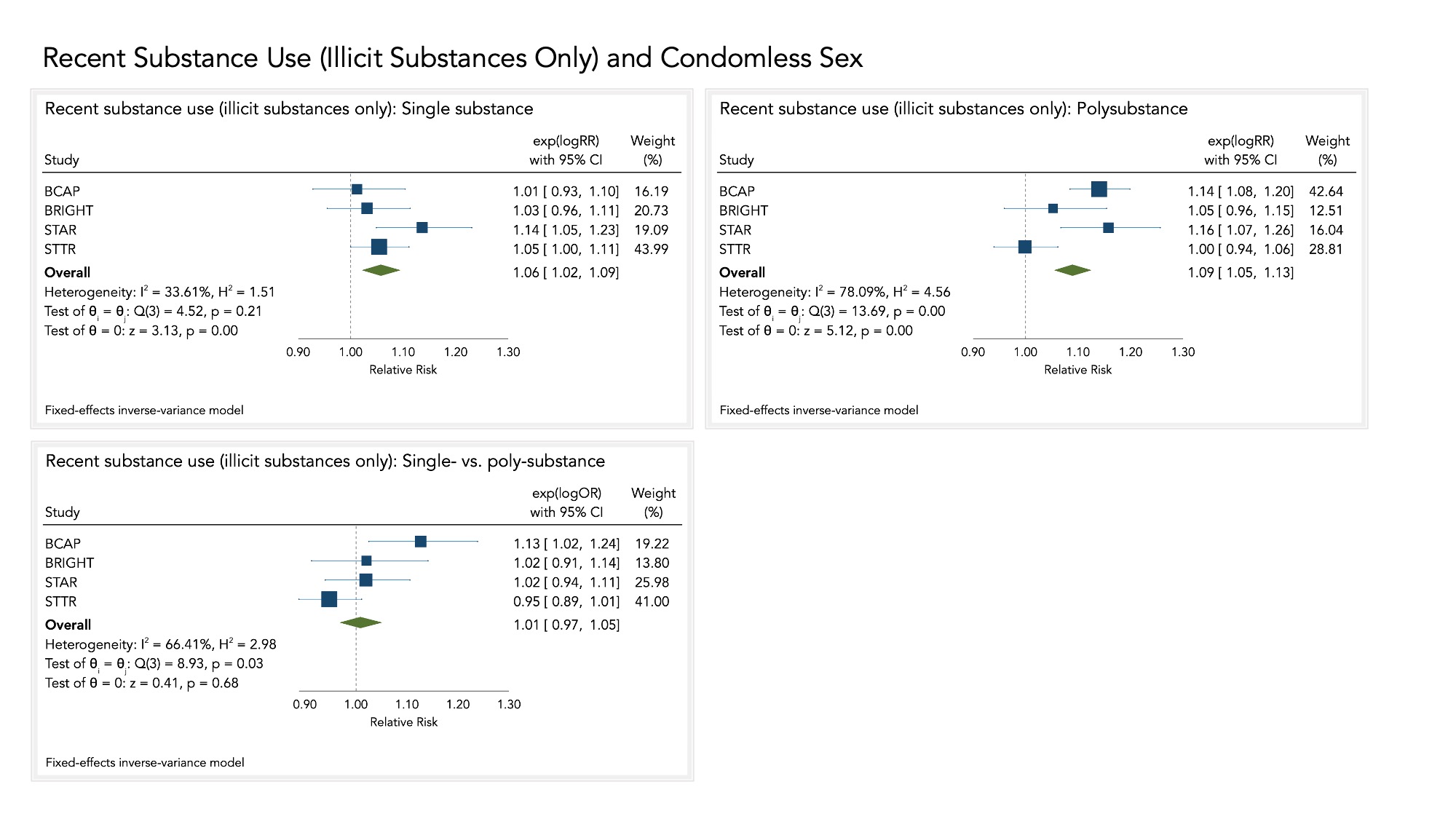


**Supplemental Figure 3**


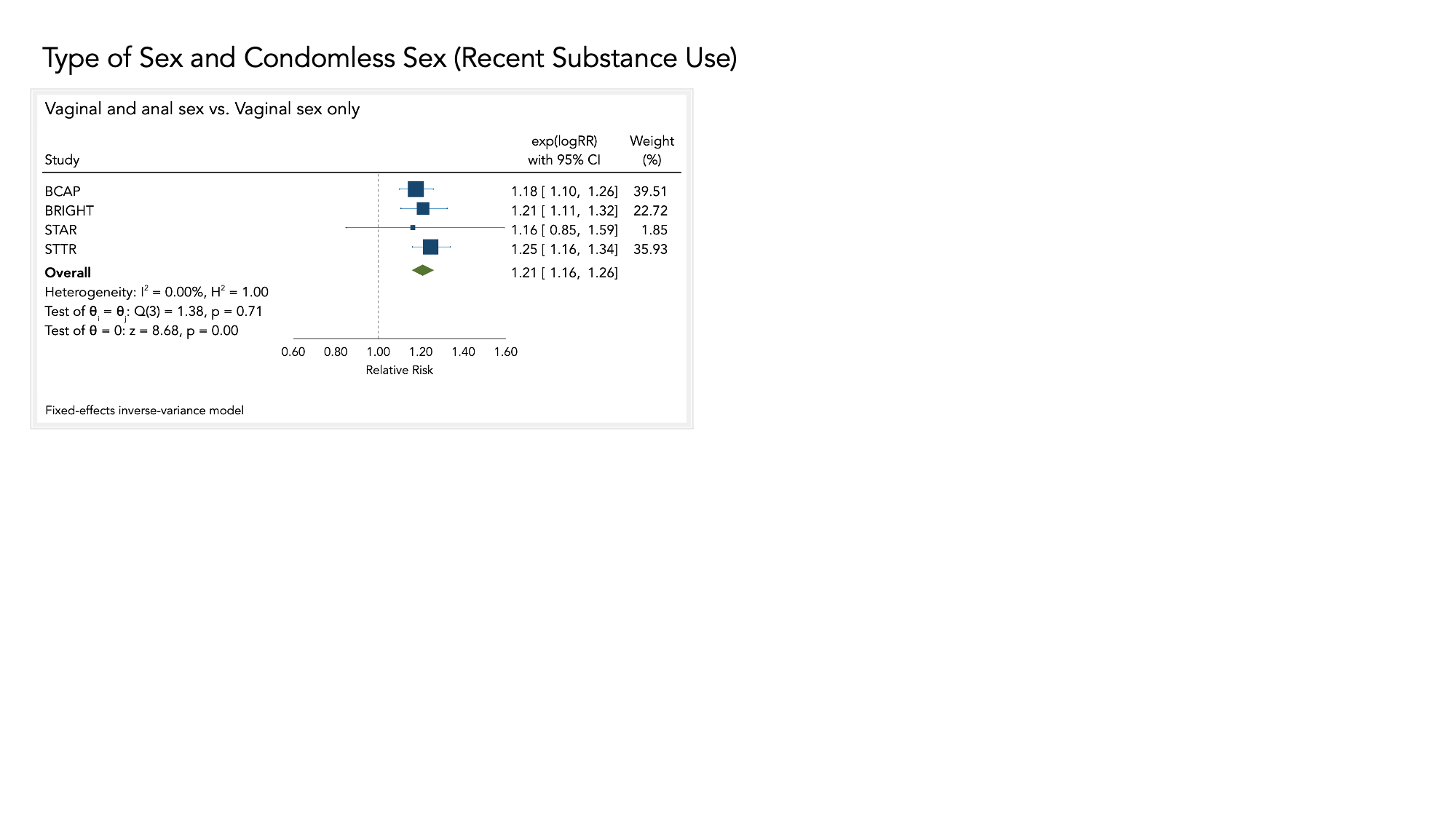


**Supplemental Figure 4**


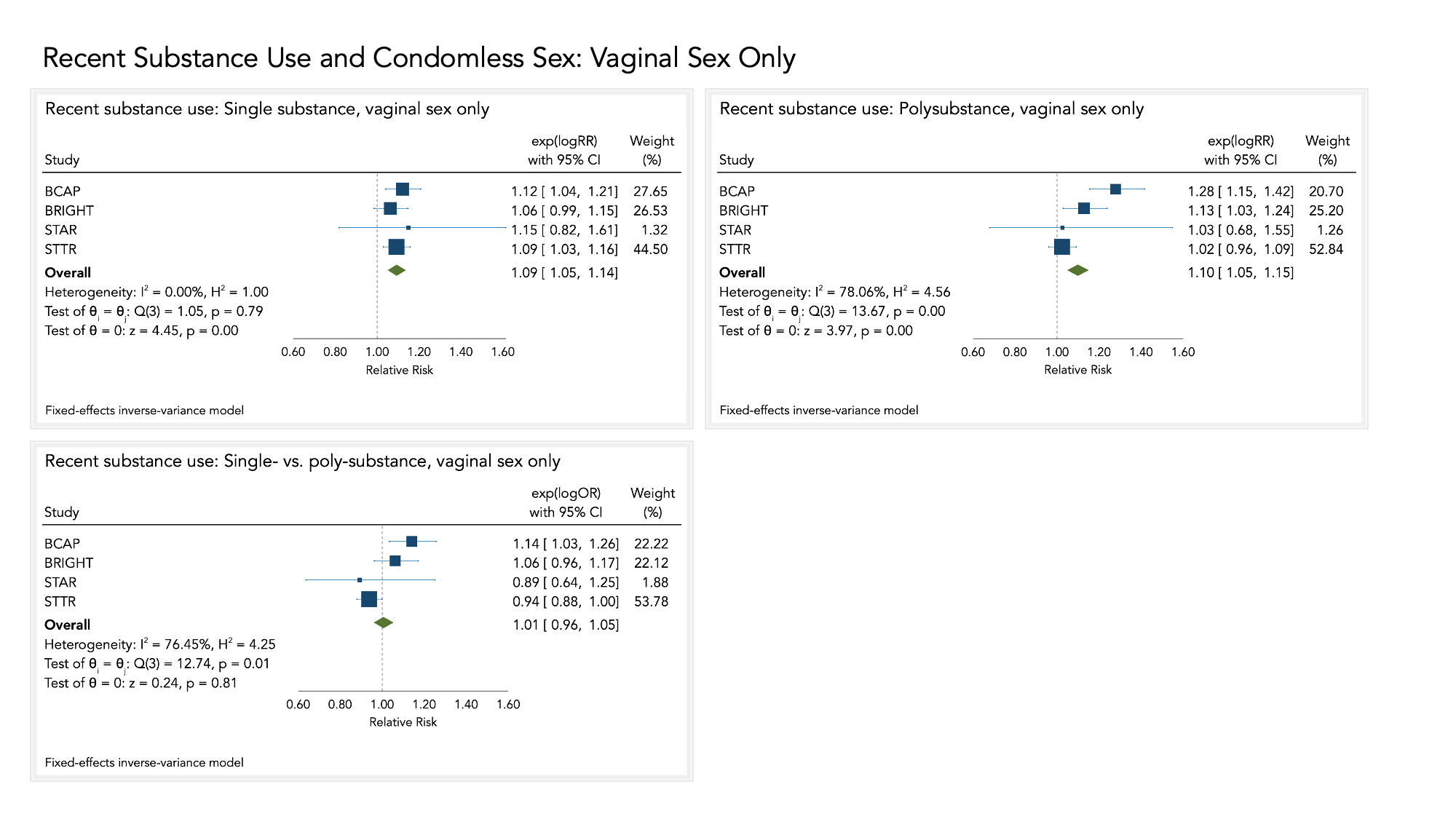


**Supplemental Figure 5**


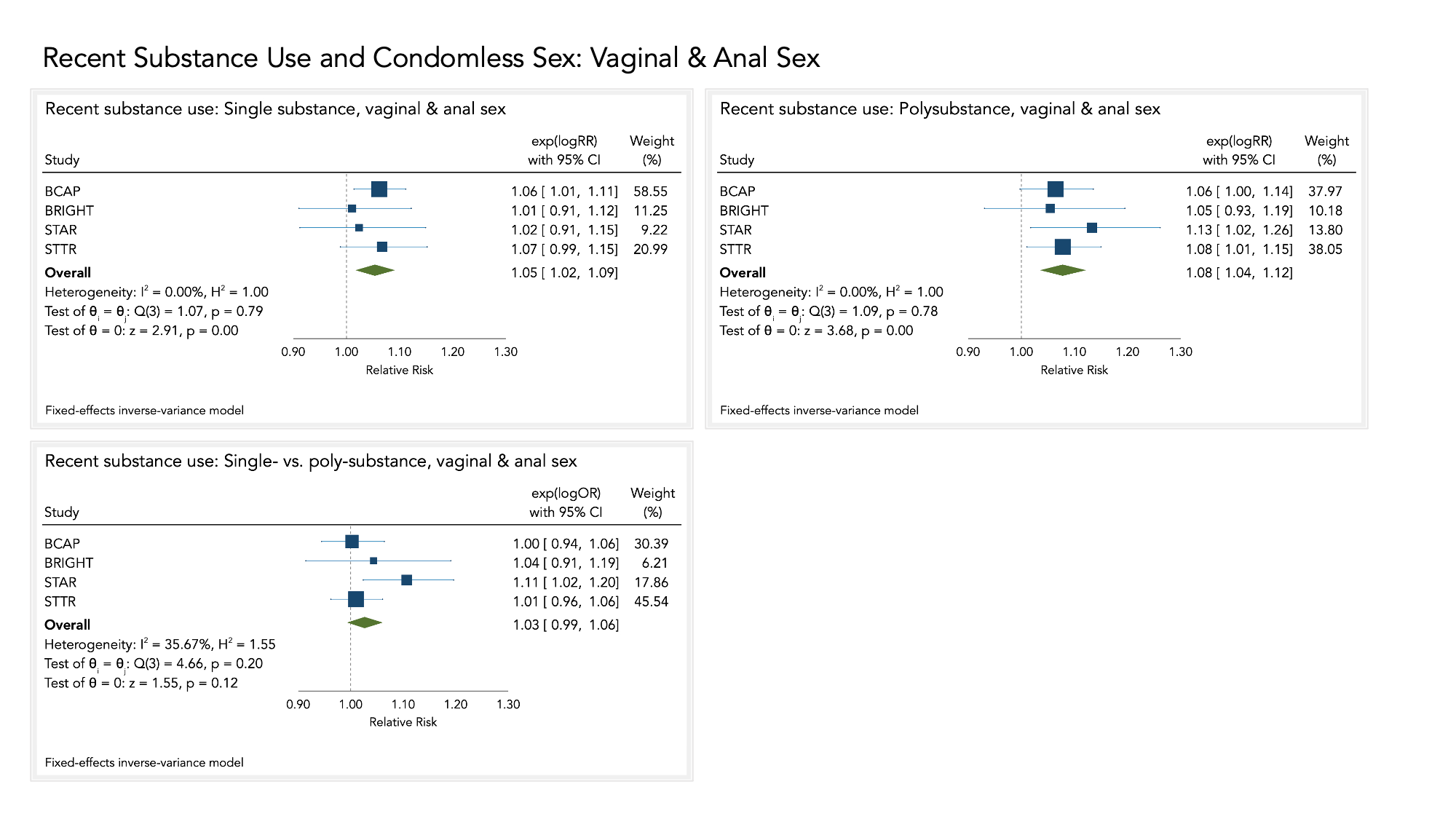


**Supplemental Figure 6**


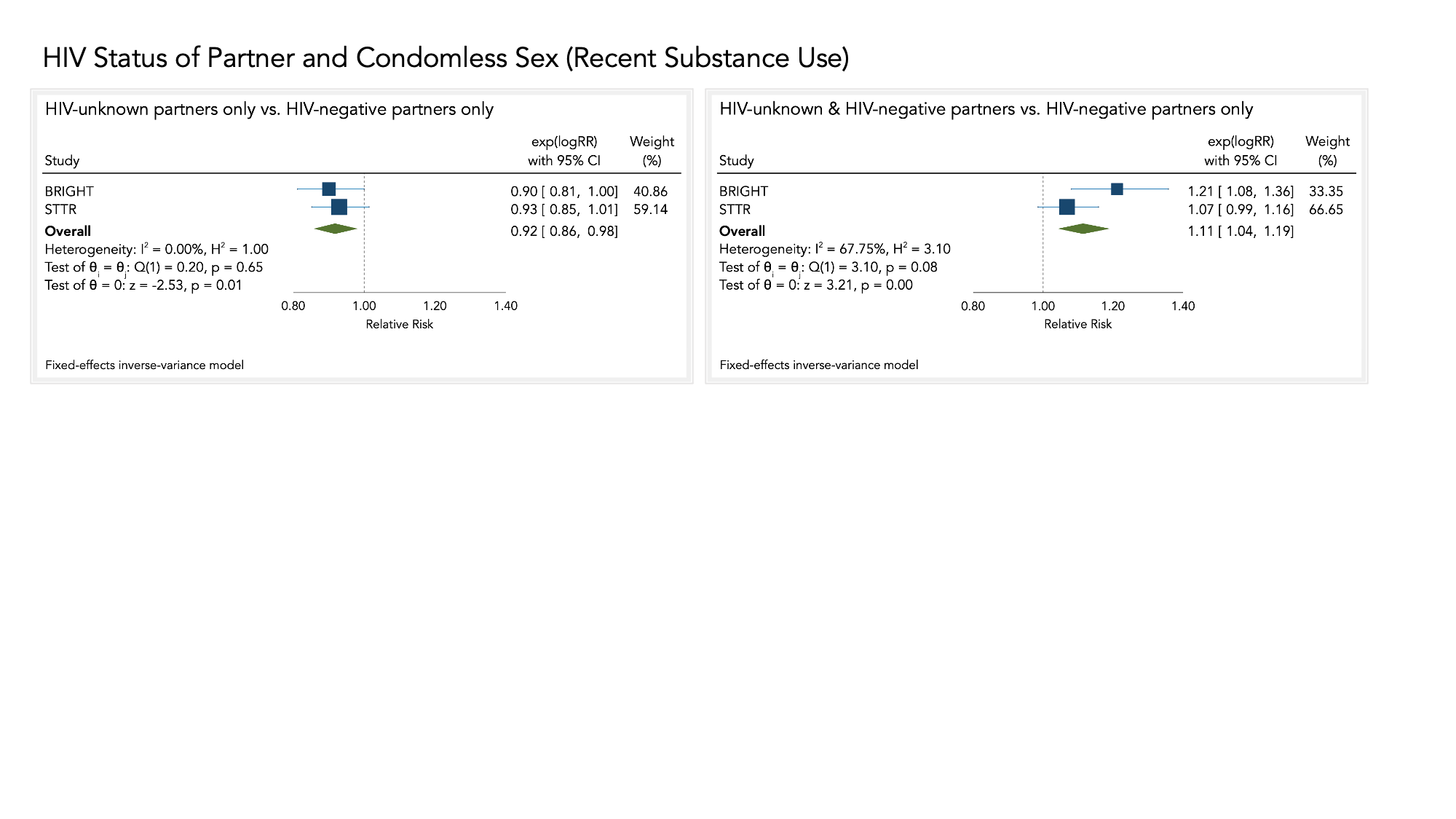


**Supplemental Figure 7**


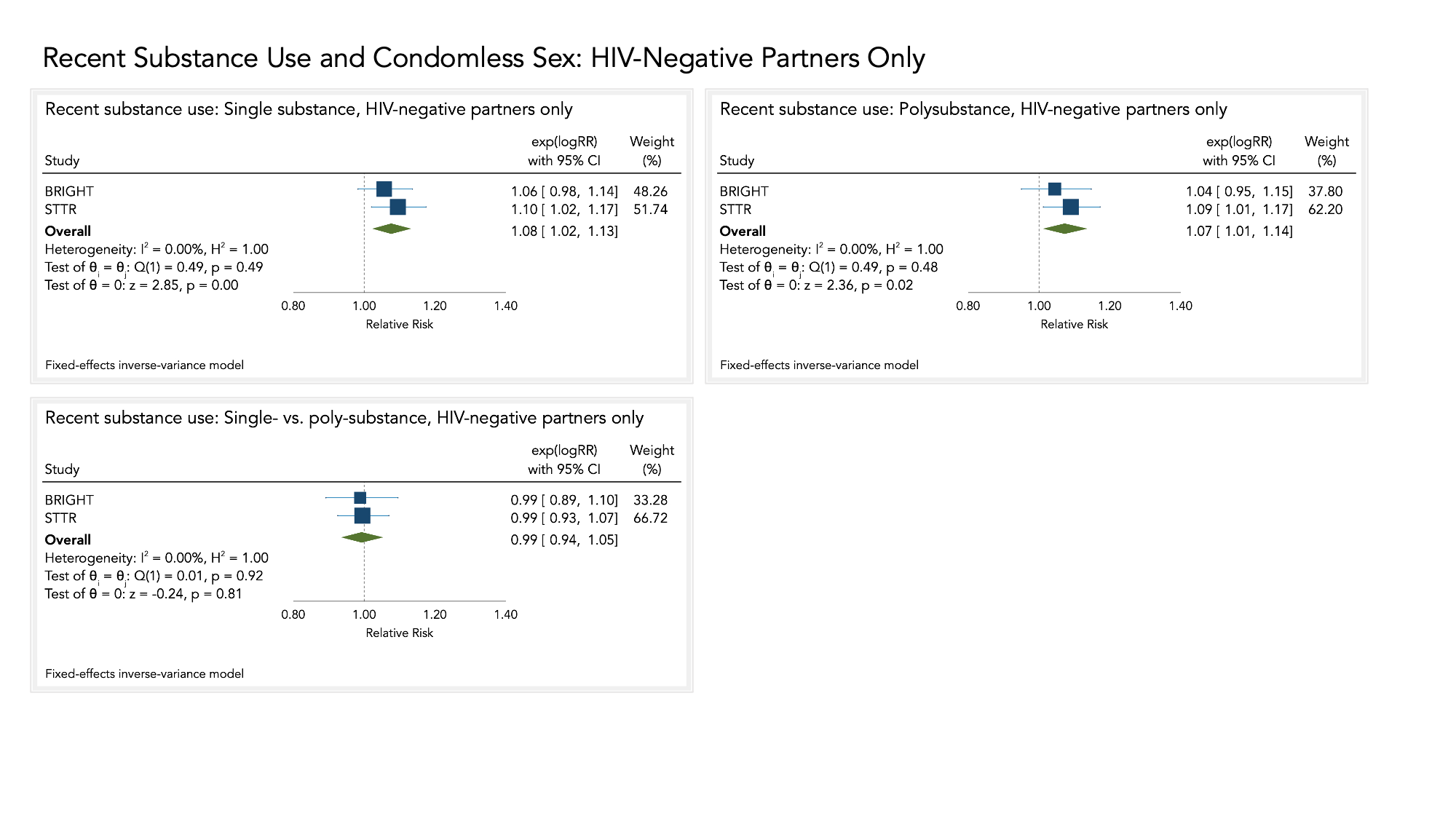


**Supplemental Figure 8**


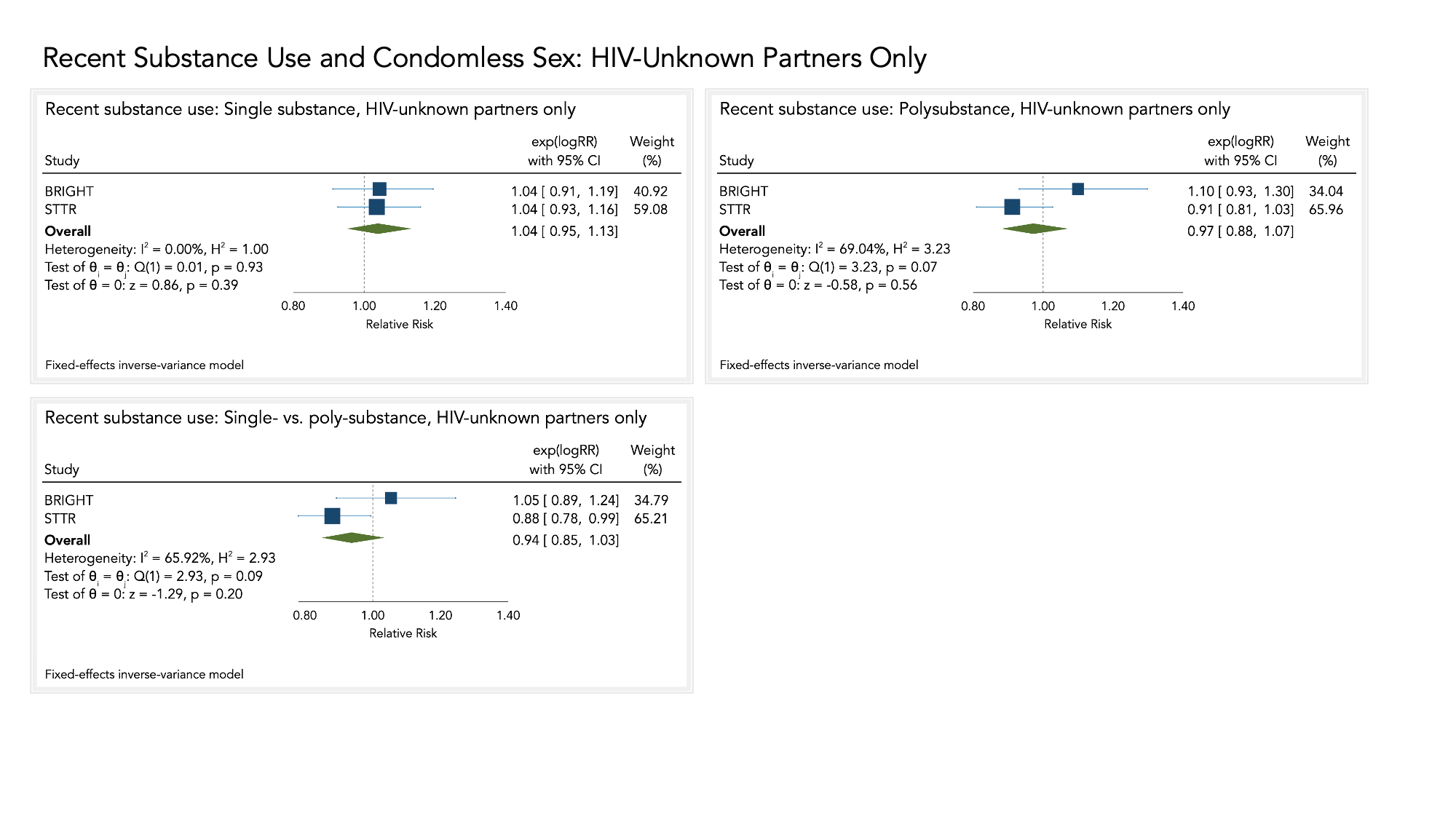


**Supplemental Figure 9**


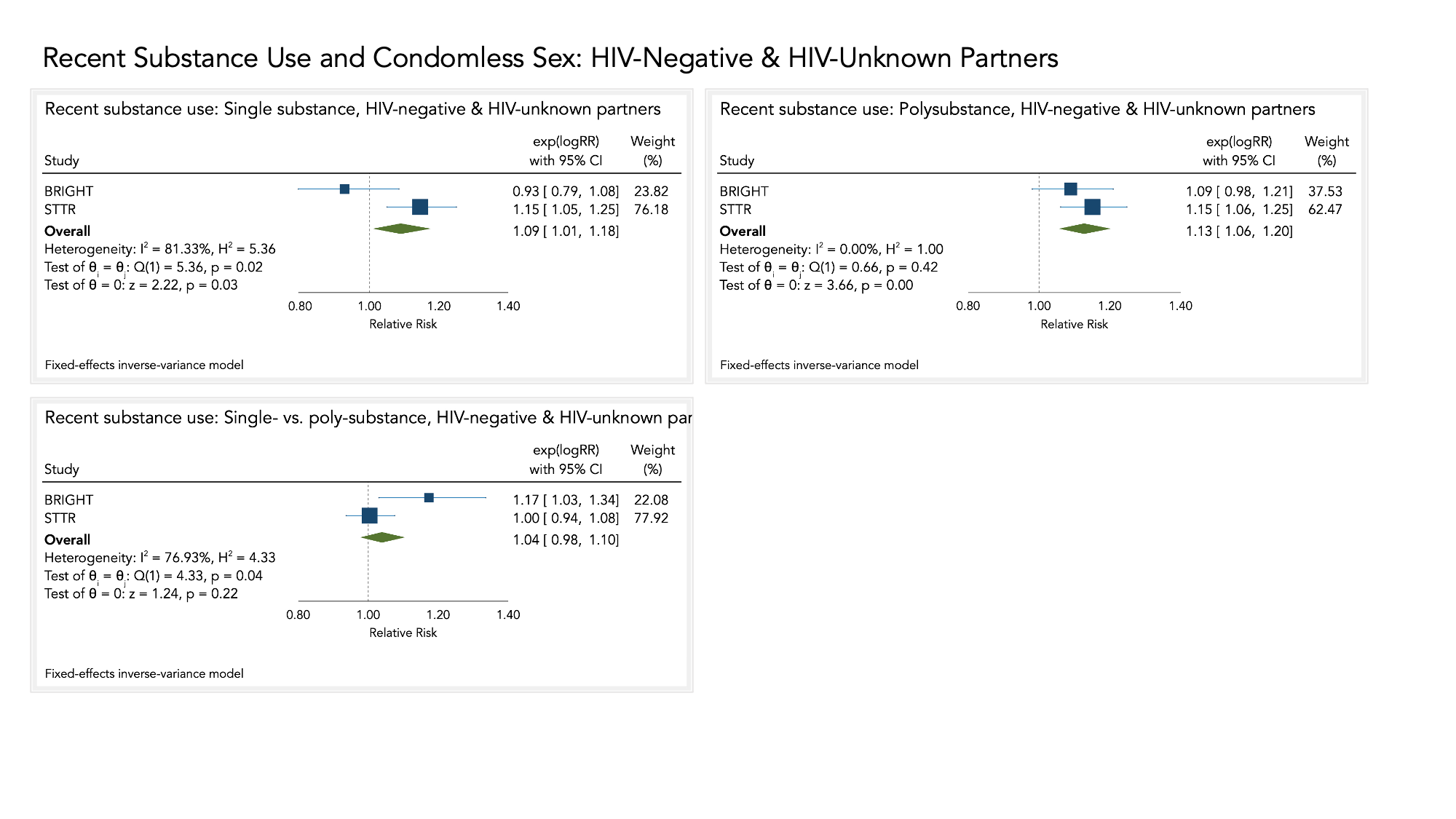


**Supplemental Figure 10**


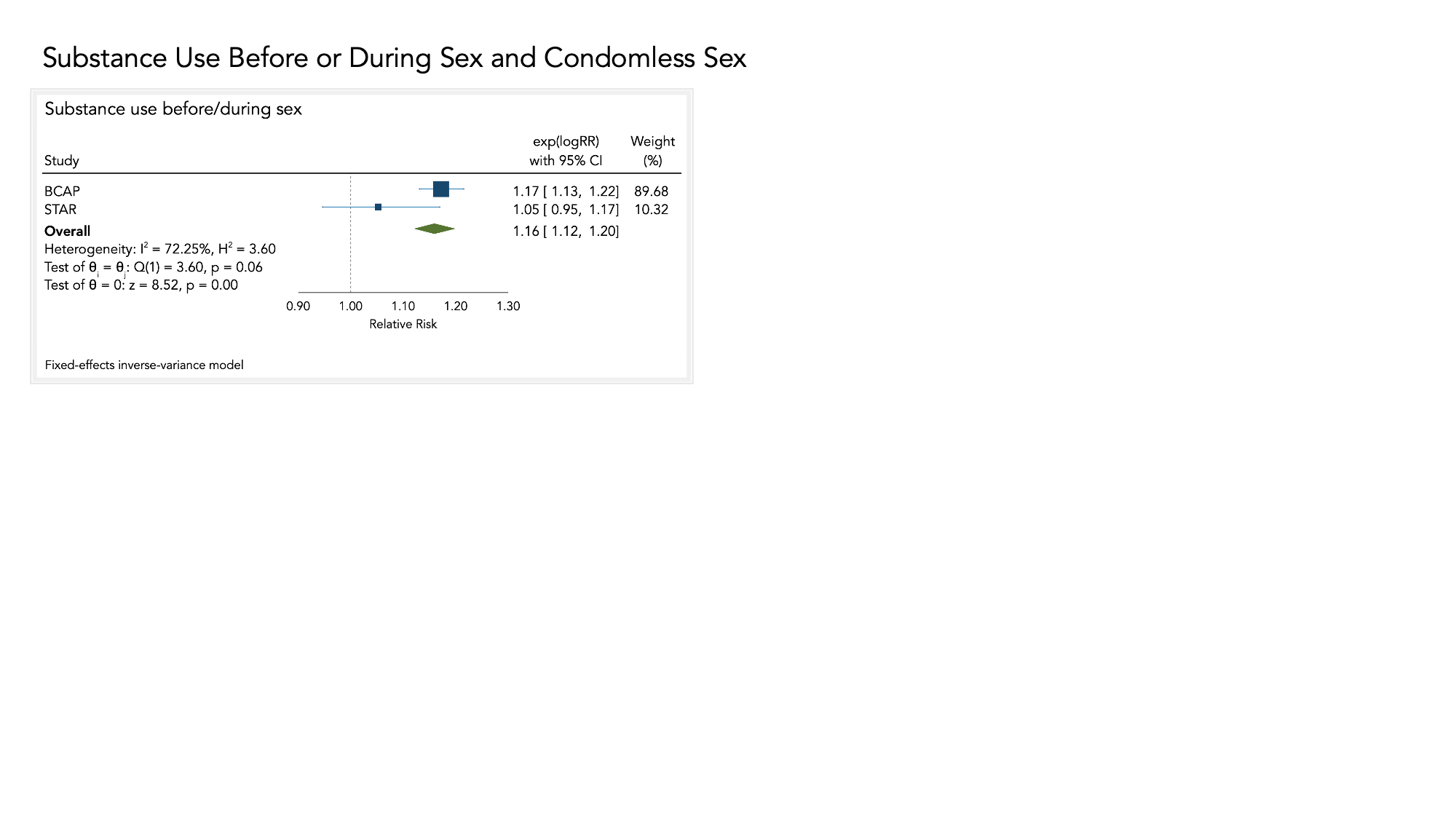


**Supplemental Figure 11**


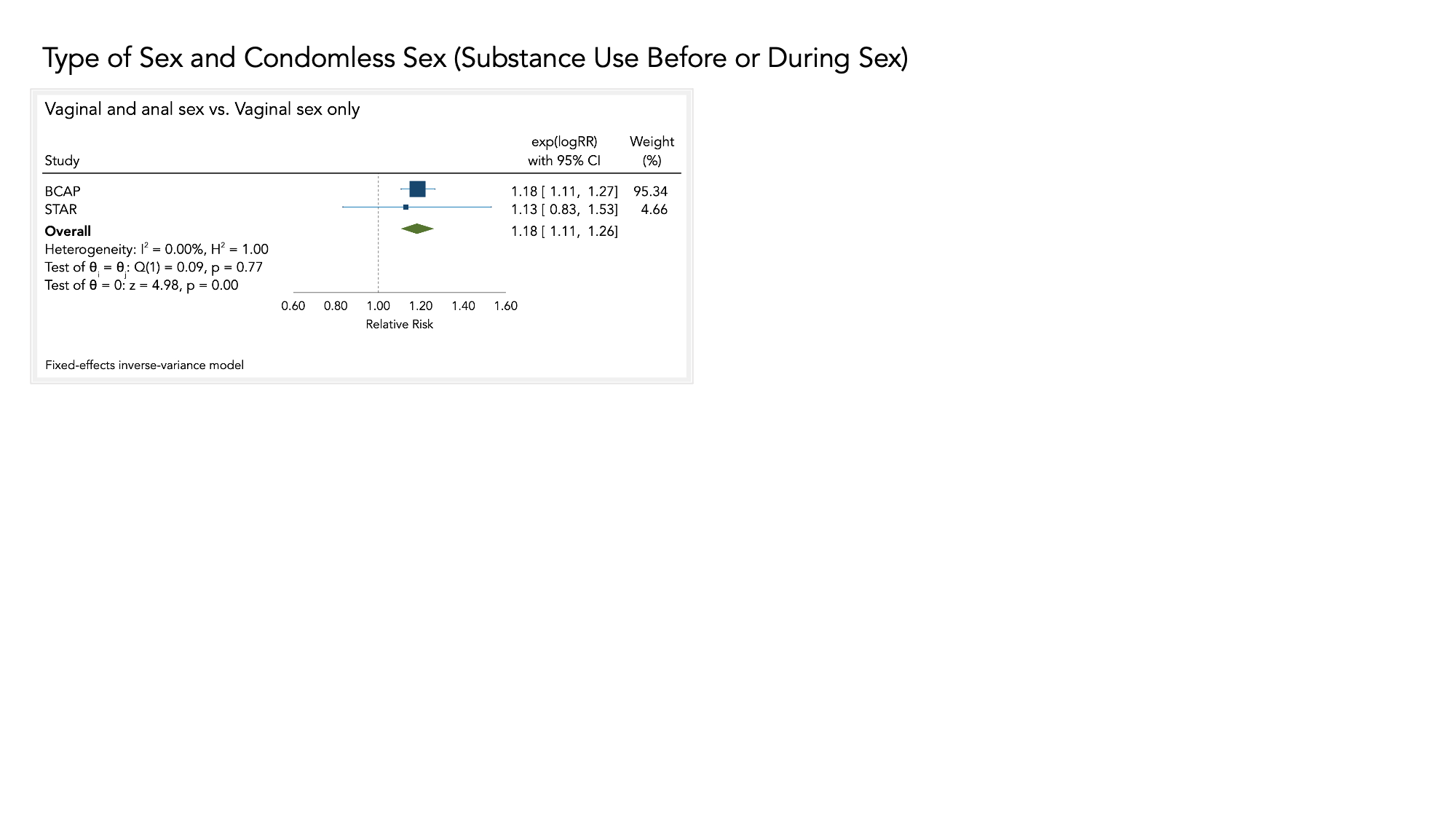


**Supplemental Figure 12**


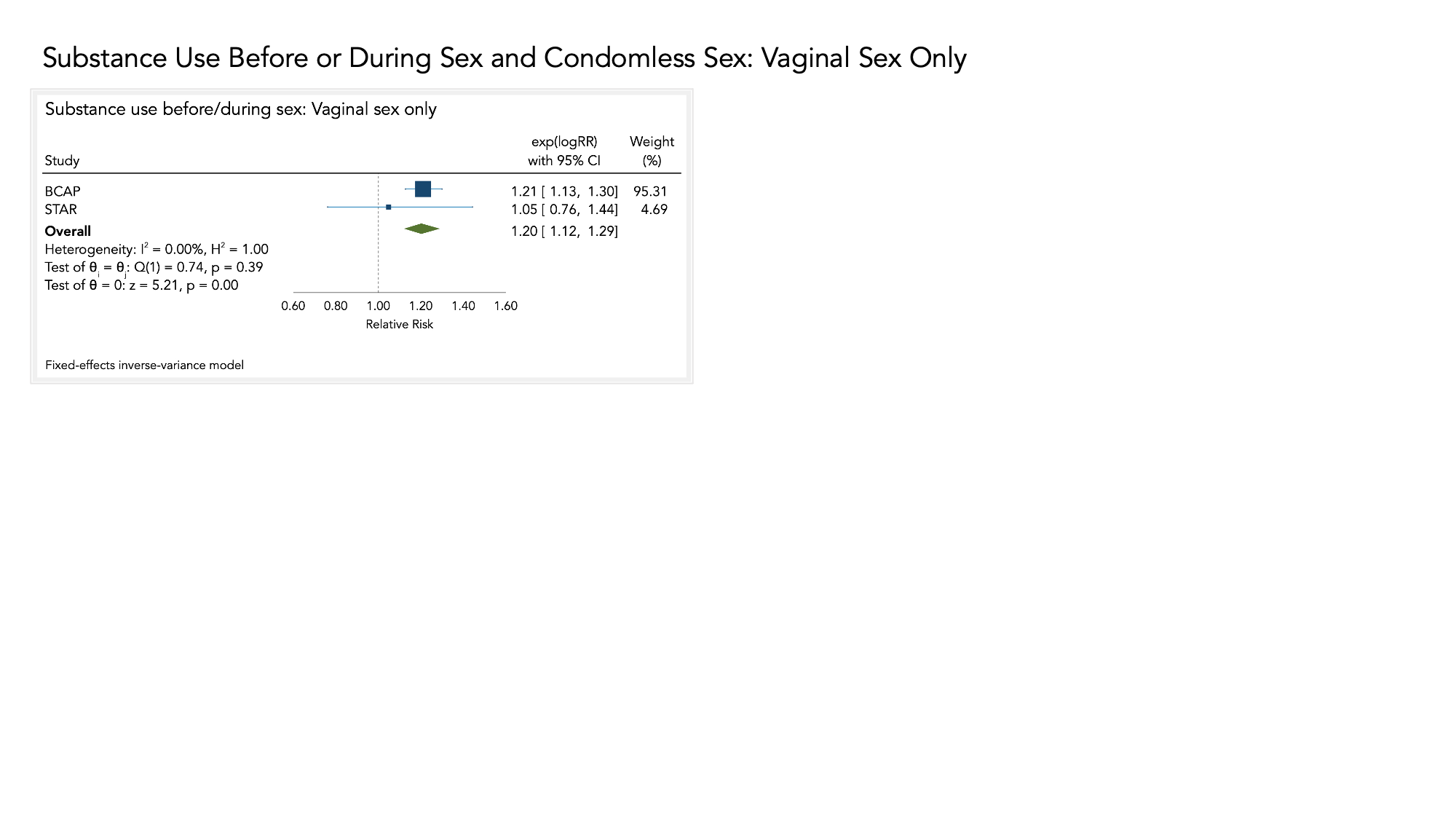


**Supplemental Figure 13**


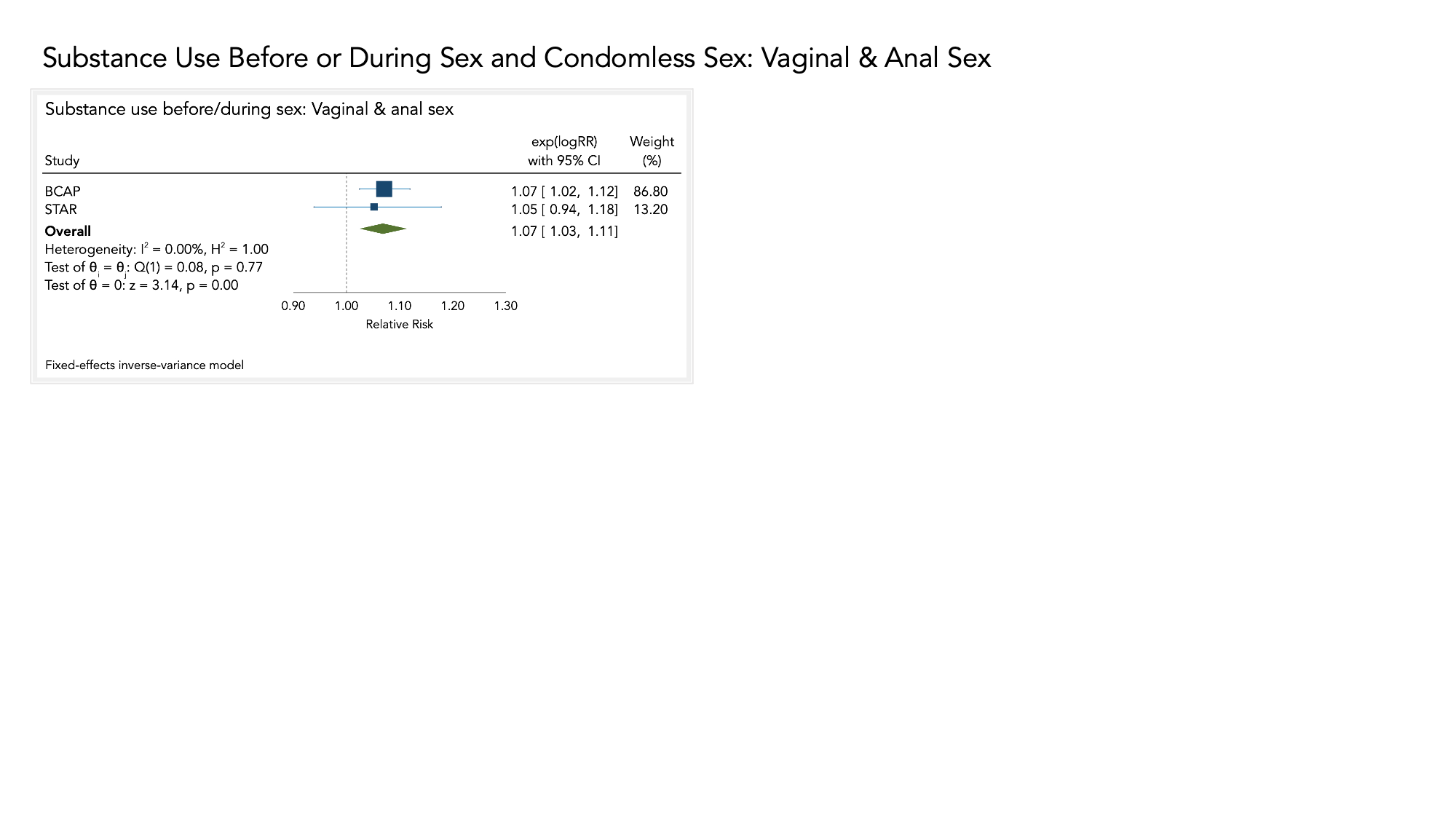

Supplement: Supplementary file 1 — Additional file 1. [file 12889_2021_12026_MOESM1_ESM.docx]
